# Epilepsy Is a Risk Factor for Sudden Cardiac Arrest in the General Population

**DOI:** 10.1371/journal.pone.0042749

**Published:** 2012-08-14

**Authors:** Abdennasser Bardai, Robert J. Lamberts, Marieke T. Blom, Anne M. Spanjaart, Jocelyn Berdowski, Sebastiaan R. van der Staal, Henk J. Brouwer, Rudolph W. Koster, Josemir W. Sander, Roland D. Thijs, Hanno L. Tan

**Affiliations:** 1 Heart Failure Research Center, University of Amsterdam, Amsterdam, The Netherlands; 2 Interuniversity Cardiology Institute Netherlands, Utrecht, The Netherlands; 3 SEIN- Epilepsy Institute in The Netherlands Foundation, Heemstede, The Netherlands; 4 Department of Cardiology, University of Amsterdam, Amsterdam, The Netherlands; 5 Department of General Practice, Academic Medical Center, University of Amsterdam, Amsterdam, The Netherlands; 6 Department of Neurology, Leiden University Medical Center, Leiden, The Netherlands; 7 Department of Clinical and Experimental Epilepsy, University College London Institute of Neurology, Queen Square, London, United Kingdom; Innsbruck Medical University, Austria

## Abstract

**Background:**

People with epilepsy are at increased risk for sudden death. The most prevalent cause of sudden death in the general population is sudden cardiac arrest (SCA) due to ventricular fibrillation (VF). SCA may contribute to the increased incidence of sudden death in people with epilepsy. We assessed whether the risk for SCA is increased in epilepsy by determining the risk for SCA among people with active epilepsy in a community-based study.

**Methods and Results:**

This investigation was part of the Amsterdam Resuscitation Studies (ARREST) in the Netherlands. It was designed to assess SCA risk in the general population. All SCA cases in the study area were identified and matched to controls (by age, sex, and SCA date). A diagnosis of active epilepsy was ascertained in all cases and controls. Relative risk for SCA was estimated by calculating the adjusted odds ratios using conditional logistic regression (adjustment was made for known risk factors for SCA). We identified 1019 cases of SCA with ECG-documented VF, and matched them to 2834 controls. There were 12 people with active epilepsy among cases and 12 among controls. Epilepsy was associated with a three-fold increased risk for SCA (adjusted OR 2.9 [95%CI 1.1–8.0.], p = 0.034). The risk for SCA in epilepsy was particularly increased in young and females.

**Conclusion:**

Epilepsy in the general population seems to be associated with an increased risk for SCA.

## Introduction

Epilepsy affects over 50 million individuals worldwide [1]. It is associated with a 2–3 fold risk of premature mortality compared with the general population [2]. A substantial proportion of deaths in epilepsy happen suddenly [3]. If trauma, drowning and a documented status epilepticus are excluded and autopsy does not reveal an anatomical or toxicological cause for death, such deaths are classified as sudden unexpected death in epilepsy (SUDEP) [4]. In the general population the commonest cause of sudden death by far is sudden cardiac arrest (SCA) due to ventricular fibrillation (VF) [5]. It has been postulated that SUDEP at least in some may result from seizure-related SCA [6]. Various possible causes for an association between epilepsy and SCA have been proposed (discussed in [7]). For instance, epilepsy and cardiac arrhythmias are both caused by pathological electrical activity, occurring in the brain and heart, respectively [8]–[10]. If either epilepsy or SCA results from ion channel dysfunction, these ion channel isoforms may be expressed both in brain and heart, as seen in *SCN1A*, which encodes neuronal sodium channels [11]. *SCN1A* mutations cause generalized seizures with febrile seizures plus [11]–[13]. Yet, *SCN1A* protein products are not only expressed in the brain, but also in the heart [14]–[15]. It is thus plausible that dysfunction in such ion channels may cause both epilepsy and SCA. It has not been proven, however, that epilepsy is associated with an increased risk for SCA, as studies aimed at systematically determining the risk for SCA among people with epilepsy are lacking.

The aim of this study was to establish whether epilepsy is associated with an increased risk for SCA. Our study was designed to obtain full coverage in the community, and provide strict confirmation of epilepsy and SCA. To achieve this, we used a mandatory multiple-source notification system for SCA. This strategy ensured the capture of all SCA cases. Every medical history was then rigorously reviewed for evidence of active epilepsy, so that all people with SCA and epilepsy were identified.

## Methods

### Setting and study design

This investigation was conducted in a community-based study in the Netherlands: the Amsterdam Resuscitation Studies (ARREST). It was designed to assess the determinants of SCA in the general community [16]–[18]. Data were retrieved in the study period July 2005–January 2010. It was conducted in accordance with the Declaration of Helsinki. Written informed consent was obtained from all participants who survived the SCA. The Ethics Committee of the Academic Medical Center Amsterdam approved the study and the use of data from people who died.

### Design of ARREST

ARREST is a prospective, community-based study aimed at establishing the genetic and clinical determinants of SCA in the population of a contiguous region (urban and rural communities, ∼2.4 million inhabitants) of the Netherlands. Details of the study design are provided elsewhere [16]–[18]. Briefly, the ARREST research group prospectively collects data of all cardiopulmonary resuscitation efforts in collaboration with all Emergency Medical Services in the region, using a mandatory multiple-source notification system (consisting of personnel at emergency dispatch centers, ambulance services and all 14 area hospitals). This ensures a complete coverage of the study region and an inclusion rate of >95% of all people with out-of-hospital SCA [16]. A data collection infrastructure is used that records all out-of-hospital SCA parameters, from ambulance dispatch to discharge from the hospital or to death. Case inclusion is as follows: after each suspected out-of-hospital SCA, the emergency dispatch center notifies the study office (providing information on the place and circumstances of SCA). Ambulance personnel are mandated to upload ECG recordings to the study office straight after resuscitation, and to provide appropriate information (e.g., whether SCA was witnessed, whether basic life support was provided before arrival of ambulance personnel, whether the patient died at the resuscitation site or was transported to a hospital). If an automated external defibrillator was used, the study center is notified by the dispatch center, ambulance personnel and the user of the automated external defibrillator (a label requesting notification after each use is attached to automated external defibrillators in the study region). The automated external defibrillator ECG recordings are retrieved by ARREST personnel after notification. ECG recordings are used to determine whether VF had occurred. SCA cases are defined as people who had a cardiac arrest in an out-of-hospital setting with ECG-documented VF. Patients with an obvious non-cardiac cause of VF (e.g., trauma, intoxication, drowning, suicide) or those in whom no ECG-documented VF was available (these patients typically had asystole or pulseless electrical activity) are excluded. Medical histories are obtained from the general practitioner (GP) and from hospital. In the Netherlands, every individual has a GP who acts as gatekeeper for medical care. Thus, GPs have a full overview of diagnoses made by medical specialists. Complete medication histories of the year preceding SCA are obtained from community pharmacies. Controls were randomly drawn from the same source community as cases, using the HAG-net-AMC database of general practitioners (GPs). This database contains the complete medical records of ∼60,000 people from a large group of GPs in the study area [19]. Each case was matched to up to 5 controls by age, sex, and index date (date of SCA in cases).

### Definition of active epilepsy and risk factors

All GP records with the terms “epilepsy” or “epileptic seizure” in the diagnosis list were reviewed by two epileptologists (RDT, JWS). For all cases and controls, epilepsy was confirmed if the diagnosis was established by a neurologist, in accordance with national guidelines [20]. Additional information was requested from the attending neurologist if needed. Only people with a diagnosis of active epilepsy were included in the analysis. Active epilepsy was defined as current treatment with antiepileptic drugs and seizure within the previous 2 years [21]. For all cases and controls, the following established risk factors for SCA were assessed: ischemic cardiovascular disease, heart failure, hypertension, diabetes mellitus, and hypercholesterolemia. This was established from GP records based on a formal diagnosis, or by use of medication.

### Statistical analysis

The relative risk for SCA associated with epilepsy was estimated by calculation of the adjusted odds ratios using conditional logistic regression analyses. Covariates that were univariately associated with SCA (at a p<0.1 level) were included in the regression analyses if they changed the point estimate of the association between active epilepsy and SCA by >5%; the only such covariates were heart failure and hypercholesterolemia. In addition, the odds ratio was calculated by including all covariates that univariately associated with SCA (at a p<0.1 level) in the multivariate analysis. Subanalyses were performed using multivariate logistic regression, adjusting for age, gender and risk factors.

## Results

We identified 1019 SCA cases with known medical and/or medication use history prior to the SCA; these cases were matched to 2834 controls. The mean age was 63.5 years in cases (76.5% male), and 58.3 years in controls (68.5% male). We confirmed that the established risk factors for SCA were also associated with SCA in our study (Table 1). Twelve cases (1.4%) and 12 controls (0.4%) had a diagnosis of active epilepsy at index date. Epilepsy was associated with an almost three-fold increased risk for SCA (adjusted OR 2.9 [95% CI 1.1–8.0], p = 0.034, model 2, Table 2). Sub-analyses suggests that SCA risk is higher in people with epilepsy aged <50 years (N = 4, adjusted OR_young_ 4.6, p = 0.210) compared to patients aged ≥50 years (N = 8, adjusted OR_old_ 2.4, p = 0.128), and in females (N = 5, adjusted OR_females_ 4.6, p = 0.044) compared to males (N = 7, adjusted OR_males_ 2.0, p = 0.309). Epilepsy characteristics of the cases and controls are given in Table 3.

**Table 1:** Demographics and distribution of covariates.

	Cases	Controls	Cases with epilepsy
	N = 1019	N = 2834	N = 12
Sex			
Male	780 (76.5)	1855 (68.5)	7 (58.3)
Female	239 (23.5)	979 (31.5)	5 (41.7)
Mean age, years (SD)	63.5 (13.7)	58.3 (14.5)	60.0 (16.0)
Covariates			
Active epilepsy	12 (1.4)	12 (0.4)	
Ischemic CVD	443 (43.5)	141 (5.0)	5 (41.7)
CVA or TIA	49 (4.8)	71 (2.5)	1 (8.3)
Hypertension	529 (51.9)	433 (15.3)	7 (58.3)
Diabetes mellitus	219 (21.5)	294 (10.4)	1 (8.3)
Heart failure	199 (19.5)	29 (1.0)	4 (33.3)
Hypercholesterolemia	290 (28.5)	170 (6.0)	6 (30.0)

Data are expressed as number (%) unless otherwise indicated. CVD, cardiovascular disease; CVA, cerebrovascular accident; SD, Standard Deviation; TIA, transient ischemic attack.

**Table 2:** Epilepsy and risk for sudden cardiac arrest.

	OR*(95% CI)	OR** (95% CI)	OR*** (95% CI)	Univariate analysis
	p-value	p-value	p-value	p-value
Epilepsy	3.3 (1.4–7.5)	2.8 (0.9–9.0)	2.9 (1.1–8.0)	Not applicable
	p = 0.005	p = 0.076	p = 0.034	
Ischemic CVD	11.2 (8.8–14.3)	6.7 (5.0–8.8)	9 (7–11.7)	p<0.001
		p<0.001	p<0.001	
CVA or TIA	1.7 (1.1–2.5)	0.8 (0.4–1.6)	Not applicable	P = 0.012
		p = 0.53		
Hypertension	5.8 (4.8–7.0)	3.7 (2.9–4.7)	Not applicable	p<0.001
		p<0.001		
Diabetes mellitus	2.0 (1.6–2.4)	0.8 (0.6–1.1)	1.2 (0.9–1.5)	p<0.001
		p = 0.1	p = 0.2	
Heart failure	20.5 (13.1–32.1)	9.9 (5.8–17)	12.9 (7.9–21.1)	p<0.001
		p<0.001	p<0.001	
Hypercholesterolemia	5.6 (4.5–7.0)	2.9 (2.2–3.9)	Not applicable	p<0.001
		p<0.001		

Abbreviations as in Table 1.

* Odds Ratios estimated with conditional logistic regression, matched on age, sex, and index date.

** Model 1: Odds Ratios estimated with conditional logistic regression, matched on age, sex, and index date, with all covariates that were univariately associated with SCA (at a p<0.05 level) included in the regression analyses (ischemic cardiovascular disease, CVA or TIA, hypertension, diabetes mellitus, heart failure and hypercholesterolemia).

*** Model 2: Odds Ratios estimated with conditional logistic regression, matched on age, sex, and index date, with covariates that were univariately associated with SCA (at a p<0.05 level) included in the regression analyses if they changed the point estimate of the association between epilepsy and SCA >5%; the only such covariates were cardiac ischemia, diabetes, and heart failure.

**Table 3:** Distribution of epilepsy and cardiovascular characteristics in cases and controls.

	Cases (n = 12)	Controls (n = 12)
Epilepsy type		
Symptomatic	8 (67%)	5 (42%)
Cryptogenic	2 (16.5%)	4 (33%)
Idiopathic	0 (0%)	2 (17%)
Unknown	2 (16.5%)	1 (8%)
Seizure type^1^		
Convulsive seizures	9	10
Complex partial seizures	3	4
Simple partial seizures	1	1
Absence seizures	1	0
Age at onset of epilepsy, yr (Median, Range)	46.5 (9–79)	42 (6–63)
Duration of epilepsy, yr (Median, Range)	11 (0–52)	17.5 (2–33)
Polytherapy (>1 antiepileptic drug)		
Yes	8 (67%)	2 (17%)
No	4 (33%)	10 (83%)
Antiepileptic drug use^1^		
Valproic acid	7	4
Carbamazepine	4	5
Phenytoin	3	2
Phenobarbital	1	1
Topiramate	1	1
Clobazam	1	0
Lamotrigine	0	2
History of underlying heart disease		
Ischemic heart disease	3 (25%)	2 (17%)
Heart failure	2 (17%)	0 (0%)
Structural heart disease^2^	3 (25%)	1 (8%)
No history	4 (33%)	9 (75%)
Cardiac medication use^1^		
Platelet aggregation inhibitors	5	1
Antihypertensives^3^	8	2
Antiarrhythmic agents^4^	1	0
Statins	5	0
Evidence of acute myocardial infarction^5^		
Postmortem^6^	3	
Clinical^7^	8	
Not available	4	

1 Some patients had more than one type of seizure/antiepileptic/cardiac drug. Therefore the number of seizure types/antiepileptic drug used exceeds the total number of patients.

2 Valve abnormalities and/or aortic coarctation.

3 Diuretics, β-adrenoceptor blockers, calcium channel blockers, angiotensin converting enzyme inhibitors, angiotensin II receptor antagonists.

4 Amiodaron.

5 Patients may fall in more than one category.

6 Evidence of acute myocardial infarction found during autopsy.

7 Evidence of acute myocardial infarction found during clinical diagnosis and treatment of the sudden cardiac arrest (ECG, cardiac enzymes, coronary angiography).

## Discussion

We provide the first systematically collected evidence from a community-based study that epilepsy in the general population is associated with an increased risk for SCA. The major strength of our study is its community-based design, which ensured that selection bias was minimal. The inclusion of people with epilepsy and of SCA was systematic. The point prevalence of active epilepsy in our control group was 0.4%. This agrees well with previous studies on the prevalence of active epilepsy in the general population and suggests that our study design captured all people with active epilepsy [22]. The design of the study and access to GPs' medical records enabled us to collect comprehensive information on circumstances surrounding death, concomitant diseases, and potential confounders.

We found that epilepsy increases SCA risk but that this excess risk is greater in the young. Given that cardiovascular diseases, by far the most prevalent causes of SCA [5], is less common in young persons, this observation supports the notion that epilepsy is likely to play an important role in SCA risk. Women also had greater excess risk than men, although in the population cardiovascular disease is more prevalent among men.

It is very difficult to study SCA in the general population given the highly unpredictable way in which it occurs, its short duration before death ensues, and its dismal survival rate [23]. ECG-documentation of VF may be the best possible method in a community-based study to ascertain that SCA was due to cardiac causes. Thus, the observed association between VF and epilepsy in ARREST supports the hypothesis that cardiac causes may contribute to SUDEP. SUDEP most frequently occurs in people with chronic epilepsy, poor seizure control, antiepileptic drug polytherapy, young age of onset and a long history of epilepsy [24]. Case-control studies, eyewitness accounts, and ictal recordings suggest that SUDEP is a peri-ictal event likely triggered by a convulsive seizure [7].

Our findings may suggest that the risk for SCA from cardiac causes extends to people with epilepsy patients beyond those with SUDEP. Firstly, SCA risk is increased in the community, i.e., in people with less severe forms of epilepsy. Secondly, in the SUDEP definition, the role of epilepsy as a risk factor for SCA may be underestimated. For instance, an individual with epilepsy who dies suddenly and has acute signs of ischemic heart disease at autopsy is not a SUDEP case, as its definition requires that clear causes for death are absent at autopsy [4]. In this person, epilepsy would be excluded from further consideration as a contributing factor to sudden death. Yet, epilepsy or its underlying pathophysiologic mechanisms may have triggered the lethal cardiac arrhythmia if the susceptibility to SCA was increased by ischemic heart disease (multiple-hit model). Accordingly, we found that, in SCA cases with epilepsy, the prevalence of known SCA risk factors was similar as in SCA cases without epilepsy (Table 1).

While our study supports the notion that SCA by cardiac arrhythmia may at least in part contribute to sudden death in epilepsy, it should be stressed that other proposed pathomechanisms, e.g., respiratory depression, cannot be excluded [4]. Experimental and clinical studies provide clues in support of a mechanistic link between epilepsy and cardiac arrhythmias [14], [15], [25]. The pathological processes that underlie both epilepsy and SCA may facilitate the occurrence of lethal cardiac arrhythmias, both in the presence or absence of seizures. In the majority of our cases (11/12) there was no evidence of seizure activity preceding SCA. Thus, sudden death in epilepsy may not be always seizure-related. Firstly, cardiac autonomic function may be impaired in both epilepsy patients [26]–[28] and SCA victims [29]–[31], and autonomic dysfunction is associated with lethal cardiac arrhythmias [27]. Secondly, cardiac repolarization disorders (e.g., prolongation or shortening of the QT interval of the ECG), another established risk factor for fatal cardiac arrhythmias [32], are found in people with epilepsy [7], [33]–[39]. Thirdly, variants in genes which encode ion channels that are expressed both in the brain and the heart may predispose for both epilepsy and cardiac arrhythmias [11], [36], [40]–[43]. Fourthly, anti-epileptic drugs may increase SCA risk by disturbing heart rhythms. For instance, they may impede cardiac conduction by blocking cardiac sodium channels, a mechanism that may trigger lethal arrhythmias in susceptible individuals, as large randomized placebo-controlled trials have indicated [44], [45]. These drugs may also act centrally affecting cardiac autonomic control. Lastly, epilepsy characteristics (type and frequency of seizures, severity of epilepsy) may modulate the risk for SCA, similar to their reported effects on SUDEP risk [7], [46]. Unfortunately, due to the small number of people with active epilepsy, we were not able to further detail the causes of excess SCA risk in epilepsy. This study may, however, serve as a basis for future studies to address these issues.

In conclusion, we provide the first systematically collected evidence that epilepsy in the community seems to be associated with an increased risk for SCA. Future studies must establish the causes of excess SCA risk in epilepsy.
