# Correction: Epilepsy Is a Risk Factor for Sudden Cardiac Arrest in the General Population

**DOI:** 10.1371/annotation/847ccbb3-45b2-4338-9ff3-ee5f35c58551

**Published:** 2012-09-26

**Authors:** Abdennasser Bardai, Robert J. Lamberts, Marieke T. Blom, Anne M. Spanjaart, Jocelyn Berdowski, Sebastiaan R. van der Staal, Henk J. Brouwer, Rudolph W. Koster, Josemir W. Sander, Roland D. Thijs, Hanno L. Tan

There were errors in the Funding section. The correct funding information is as follows: HLT was supported by the Netherlands Organization for Scientific Research (NWO, grant ZonMW Vici 918.86.616), and the Dutch Medicines Evaluation Board (MEB/CBG). HLT, RDT, and JWS are supported by the National Epilepsy Foundation Netherlands (project number 10-07). The Arrest data collection was supported by an unconditional grant from Physio Control and a grant from the Netherlands Heart Foundation (grant 2006B179). Dr. Bardai was supported by the Netherlands Organization for Scientific Research (NWO, grant Mozaiek 017.003.084). RD Thijs and JW Sander are part of PRISM - the Prevention and Risk Identification of SUDEP Mortality Consortium which is funded by the NIH (NBIH/NINDS -1P20NS076965-01). The funders were not involved in: design, conduct of the study, collection, management, analysis, interpretation of the data, preparation, review or approval of the manuscript. 

